# Supplementary material for: Correlation analysis of mitochondrial DNA maintenance-related genes with HCC prognosis, tumor mutation burden and tumor microenvironment features
Source: PLoS One. 2025 Jun 2;20(6):e0325033. doi: 10.1371/journal.pone.0325033 (PMC12129190; doi:10.1371/journal.pone.0325033)
Supplement: S1 File — (DOCX) [file pone.0325033.s001.docx]

**Supplementary materials**

**The performance of oPLS-DA model was assessed by R²Y (goodness of fit) and Q² (goodness of prediction) through cross-validation, along with permutation testing to evaluate robustness.**

The performance of the oPLS-DA model was assessed as follows:

**1. Data correction (before/after removal of batch effect)**

Problem: Different data sets (such as TCGA and GSE76427) have a "batch effect" due to differences in experimental conditions, resulting in false positive results.

The solution: Before correction: 89.7% explained by Dim1 (dominated by technical differences). After correction: Dim1 decreased to 18.9% (biological differences highlighted).

Significance: To eliminate technical interference and ensure that the differences found later truly reflect biological characteristics.

**2. Data integration (oPLS-DA score chart)**

Key points: Integrating multiple omics data (gene expression + clinical typing).

Graphic interpretation: Horizontal/vertical axis: principal components t1 (3%) and t2 (16%).

Sample distribution: TCGA (red) and GSE76427 (blue) partially overlap but are generally separable

(please see Figure S1 C and D).

Significance: It proves that data from different sources can cooperatively reveal biological laws.

**3. Model efficacy (model efficacy -Rplot)**

Core indicators:

R2Y=0.767: The model can explain 76.7% of the sample classification differences.

Q2Y=0.672: 67.2% cross-validation prediction ability (>0.5 is valid).

p<0.05: The possibility of random guessing is excluded by substitution test.

Significance: The model is both reliable (high R2Y) and practical (high Q2Y).

**4. False positive control (replacement test chart)**

Method: Randomly scramble the labels 100 times and compare the real model (red line) with the random result (gray bar).

Result: The real R2Y/Q2Y was significantly higher than the random distribution (p=0.05).

No "virtual high" performance occurred.

Significance: Tat the differential genes and pathways discovered are not accidental.

In summary, our analytical process is rigorous (from data cleansing to model validation), and the biomarkers and typing results found have biological significance and potential clinical application.

**The specific flow chart is as follows**

**
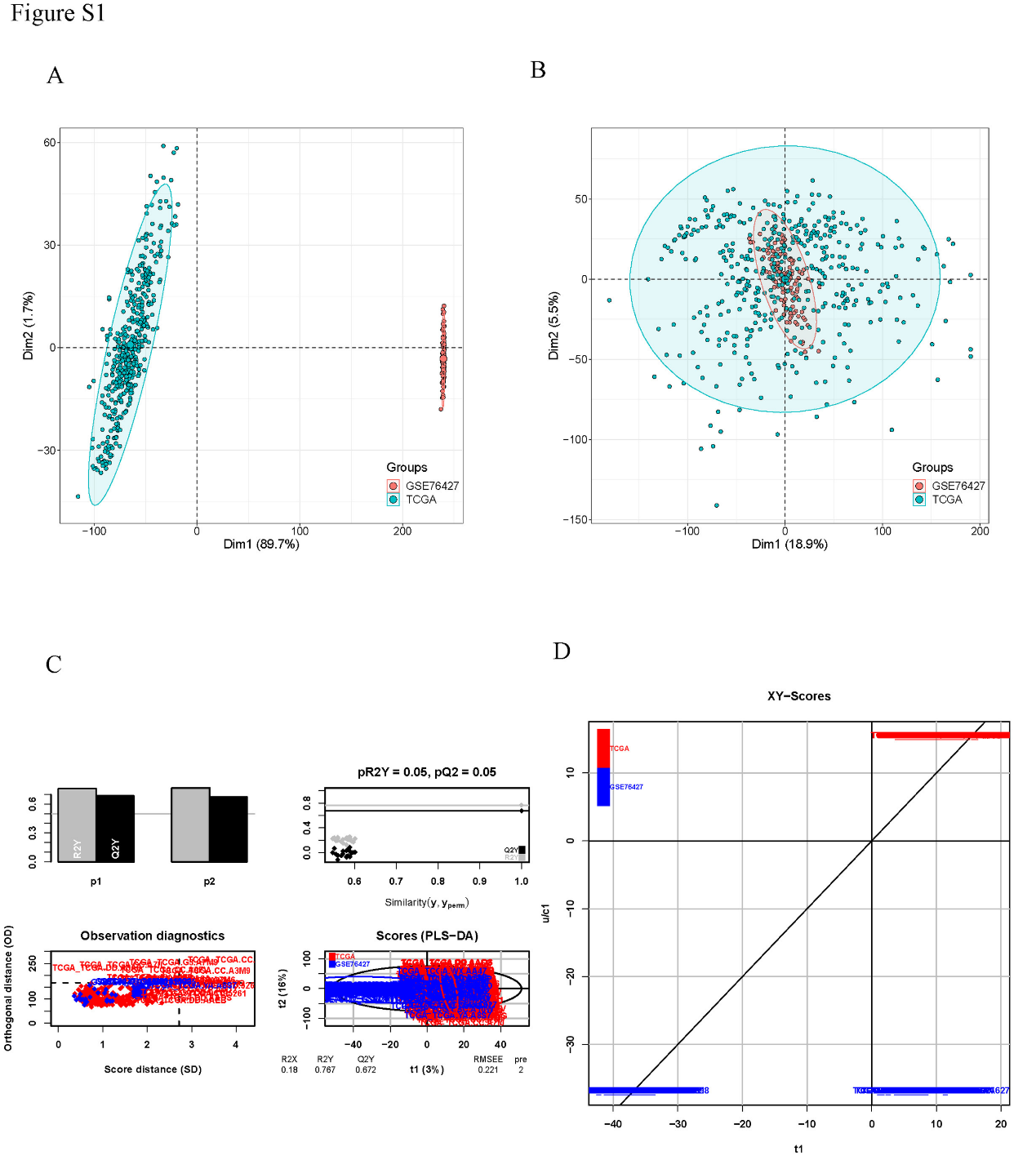
**

**Figure S1. Data set of HCC samples before and after batch correction.** A. Batch of HCC samples before correction. B. Batch of HCC samples after correction. C. Data integration (oPLS-DA score Chart). D. Model efficacy (model efficacy -Rplot).
