# Supplementary material for: Correlation analysis of mitochondrial DNA maintenance-related genes with HCC prognosis, tumor mutation burden and tumor microenvironment features
Source: PLoS One. 2025 Jun 2;20(6):e0325033. doi: 10.1371/journal.pone.0325033 (PMC12129190; doi:10.1371/journal.pone.0325033)
Supplement: Attachment — Submitted filename: Response to the reviewers.docx [file pone.0325033.s002.docx]

**Dear Editor and Reviewers:**

Thank you very much for your efforts to review our manuscript entitled “**Correlation analysis of mitochondrial DNA maintenance-related genes with HCC prognosis, tumor mutation burden and tumor microenvironment features**”. To address the comments and concerns the Editor and Reviewers have raised, we have included a point-by-point response to each comment. Within the revised manuscript, changes to the text in response to the comments were marked as red font. We have already upload the data set to figshare, doi: 0.6084 / m9 figshare. 27089542.

We appreciate the suggestions and comments by the Editor and the Reviewers. As a consequence of these valuable suggestions, we believe that our manuscript has been much strengthened. We hope that the revised manuscript will find your consideration now.

Yours sincerely,

Zhongji Meng

Department of Infectious Disease, Taihe Hospital, Hubei University of Medicine

No. 32. South Renmin Road, Shiyan 442009, China.

E-mail: [zhongji.meng@163.com](mailto:zhongji.meng@163.com)

**Page No.**

**Response to** **Editor ……………………………………………………………………………….2**

**Response to Reviewer 1 ………………………………………………………………………… 6**

**Response to Reviewer 2 ………………………………………………………………………… 11**

**Response to Editor**

Thanks for your kind suggestions. We have read your suggestions carefully and try our best to give the following response:

**Q1.** Please define the mtDNA score, TMB and TME (lines 25-17).

**Response:** Thank you for your suggestions. The tumor microenvironment (TME) was defined (lines 73-75 in the revised manuscript). mtDNA score was defined (lines 185-186 in the revised manuscript). Tumor mutational burden (TMB) reflects cancer mutation quantity (doi:10.1016/j.ccell.2020.10.001), now TMB is emerging as a potential biomarker (doi:10.1093/annonc/mdy495). As a common name, in many articles, TMB is directly cited (doi: 10.1016/j.ccell.2023.09.006; doi:10.7150/thno.52717).

**Q2.** I suggest you use the keyword mitochondrion and not mitochondrial (line 44).

**Response:** Thank you for your suggestion. the key word “mitochondrial” has been replaced with “mitochondrion”.

**Q3.**“own superhelical double-linked 61 ring of genetic material” is unnecessary (line 60)

**Response:** Thank you for your suggestion. The sentence “A unique feature of mitochondria is that they have their own superhelical double-linked ring of genetic material, called mitochondrial DNA (mtDNA)” has been deleted.

**Q4.** Move gene list from lines 115-118 to a separate table.

**Response:** Thank you for your suggestion. If we move gene list to a separate table, there's not much in this table. So, we categorized these genes according to their functions. Maybe it's more categorical and concise. mtDNA replication (DNA2, EXOG, LIG3, POLG, POLG2, POLRMT, RNASEH1, SSBP1, TFAM, TFB2M, TOP1MT, TOP3A, TWNK)，mtDNA repair (APEX1, EXOG, LIG3, OGG1, POLG, PRIMPOL, RECQL4, UNG), mtDNA modifications (METTL4), mtDNA stability and decay (ENDOG, EXOG, MGME1) (lines 125-128 in the revised manuscript).

**Q5.** Describe ssGSEA in more detail in the Methods section.

**Response:** Thank you for your comments. The following sentence has been added the Methods section to describe ssGSEA in more detail. ssGSEA was used to calculate the standardized enrichment score via the GSVA package50.The relative abundance of each immune cell type was represented by an enrichment score in ssGSEA analysis and normalized to unity distribution from 0 to 1(doi: 10.7150/thno.52717)(lines 167 to 169).

**Q6.** What is this data set from Charoenteng? Add a reference to it.

**Response:** Thank you for your suggestion. Pornpimol Charoentong, et al analyzed tumor-immune cell interactions in 20 solid cancers, revealing the relationship between genotype and immunophenotype and created The Cancer Immunome Atlas (https://tcia.at/). Using machine learning, the determinants of tumor immunogenicity were identified and a quantitative scoring scheme called immunophenotypic scoring was developed. The according reference (doi: 10.1016/j.celrep.2016.12.019) has been added (line 156).

**Q7.** Why was an adjusted p-value of 0.001 used? (line 143)

**Response:** We referred to many similar literatures and found that using an adjusted p-value of 0.001 is more reasonable (doi: 10.1186/s12943-020-01170-0; doi: 10.3389/fcell.2022.842220).

**Q8.** Lines 166-176: indentation is problematic

**Response:** Thank you for your comment. All the indentation has been deleted in the revised manuscript.

**Q9.** you mention 8293 DEGs between clusters A and B and 2043 DEGs between clusters A and C, and 9064 between clusters B and C. Why are there so many? What was the fold change cutoff limit? What would happen if you increase this cutoff limit?

**Response:** Although the consensus clustering algorithm based on mtDNA MRGs expression classified HCC patients into three mtDNA MRG phenotypes, the underlying genetic alterations and expression perturbations within these phenotypes were not well known, so there are so many related genes. P-values were adjusted according to the false discovery rate (FDR), and the P-value less than 0.001 was taken as the screening criteria. If we increase this cutoff limit, more DEGs related genes will be excluded.

**Q10.** Starting from line 317 you describe the role of TP53 in cancer. This is well known. What is the novelty of your findings? As you write, HCC is very heterogeneous, what other genes play a role in HCC that you found?

**Response:** The novelty of our findings lies in the following aspects. 1) In the high mtDNA score group, TP53 mutation frequency is the highest, and the poor prognosis of HCC patients in the High mtDNA score group may be related to the high TP53 mutation. 2) Among HCC patients with lower mtDNA scores, HCC patients with TP53 mutations had worse clinicopathological features compared to CTNNB1-mutated HCC patients.

Besides TP53, two genes RECQL4 and APEX1 have been found play a role in HCC, HCC patients with higher expression of RECQL4 and APEX1 have a worse prognosis according with the previous studies (doi:10.3892/ol.2018.78600; doi:10.1016/j.immuni.2019.12.011). In our data, RECQL4 and APEX1 are highly expressed in HCC samples, and the higher expression in RECQL4 and APEX1, the worse prognosis of the HCC patients, which is consistent with the results of other studies. So, our research is feasible.

Response to Reviewer 1

**Reviewer 1**

Methodological Considerations: The study design appears sound, utilizing a large sample size and robust bioinformatic tools. However, there are several areas that could be improved upon for greater clarity and rigor:

**Q1.** Sample Selection and Control Group: While the use of TCGA and GEO datasets is commendable, the inclusion criteria for selecting HCC samples should be more clearly described. Moreover, the control group's characteristics should also be provided for comparison purposes.

**Response:** Dear reviewer, thanks for your kind suggestions. HCC samples in TCGA were collected from patients pathologically diagnosed with hepatocellular carcinoma (HCC) after surgical resection and who had not previously received treatment (ablation, chemotherapy, or radiotherapy) specific to their disease. Cases were staged according to the American Joint Committee on Cancer (AJCC). Each frozen primary tumor specimen had a companion normal tissue specimen (blood or blood components, including DNA extracted at the tissue source site). Adjacent tissue was submitted for some cases. Hematoxylin and eosin (H&E) stained sections from each sample were subjected to independent pathology review to confirm that the tumor specimen was histologically consistent with the allowable hepatocellular carcinomas and the adjacent tissue specimen contained no tumor cells. Adjacent tissue with cirrhotic changes was not acceptable as a germline control, but was characterized if accompanied by DNA from a patient matched blood specimen. The percent tumor nuclei, percent necrosis, and other pathology annotations were also assessed. Tumor samples with ≥ 60% tumor nuclei and ≤ 20% or less necrosis were submitted for nucleic acid extraction (doi:10.1016/j.cell.2017.05.046). GSE76427 cohort with 115 HCC samples was downloaded from the GEO database as a validation cohort after the removal of normal tissue samples and tumor samples without follow-up and outcome status information.115 primary HCC tissues and 52 adjacent tissues (Dataset GSE76427, Eastern population) from GEO were enrolled in this study. Percentage of HCC patients with HBV infection and cirrhosis were 46% and 54%, respectively (doi:[10.1016/j.heliyon.2023.e14460](https://doi.org/10.1016%2Fj.heliyon.2023.e14460);doi:[10.1002/1878-0261.12153](https://doi.org/10.1002%2F1878-0261.12153)). All HCC samples were downloaded from TCGA and GEO. The inclusion criteria for selecting HCC samples and control group's were HCC patients with survival information in the databases.

**Q2.** Screening of mtDNA MRGs: The process of identifying the 22 mtDNA MRGs requires further detail. How were these genes prioritized over others? What criteria were used for screening?

**Response:** Thank you for your suggestions. The process of identifying the 22 mtDNA MRGs was described in more detail as “The list of mtDNA MRGs was obtained from the MitoCarta3.0 database, including 27 mtDNA MRGs for mtDNA replication (DNA2, EXOG, LIG3, MGME1, POLG, POLG2, POLRMT, RNASEH1, SSBP1, TFAM, TFB2M, TOP1MT, TOP3A, TWNK)，mtDNA repair (APEX1, EXOG, LIG3, OGG1, POLB, POLG, PRIMPOL, RECQL4, UNG), mtDNA modifications (METTL4), mtDNA stability and decay (ENDOG, EXOG, MGME1), respectively. The expression profile of these genes was systematically extracted and analyzed in normal and tumor samples. Finally, 22 mtDNA MRGs with statistical significance were identified, with the function of not only packaging mitochondrial DNA, but also providing a stable environment for mitochondrial DNA replication, transcription and repair, so that they are superior to other mtDNA MRGs (doi:10.1016/j.biochi.2013.09.017).

**Q3.** Unsupervised Clustering: Details about the machine learning model employed for unsupervised clustering need to be elaborated upon, including parameters and validation methods.

**Response:** Thank you for your suggestions. Unsupervised Clustering was performed using the R package“Consensus Cluster Plus” and 1000 cycles were undertaken to ensure the stability of the classification; the number of cluster k values were increased from 2 to 9. The k = 3 with better clustering stability were selected according to the clustering effect (doi: 10.1093/bioinformatics/btq170), the detailed information has been added to the revised manuscript.

**Q4.** PCA Model Construction: The construction of the mtDNA score model using PCA should be detailed, including how the principal components were selected and validated.

**Response:** Thank you for your suggestions. In this study, the mtDNA score model was constructed using PCA refer to previous studies, the overlapping DEGs identified from different mtDNA clusters were selected and employed to perform prognostic analysis for each gene using a univariate Cox regression model. The genes with a significant prognostic impact were extracted for further feature selection by using recursive feature elimination (RFE) with random forest and the 10-fold cross -validation method in the‘caret’package. Then the expression profile of the final determined genes was curated to perform PCA analysis, and principal components 1 and 2 were extracted and served as the signature score. This method mainly focuses on the score on the set with the largest block of well correlated (or inverse-correlated) genes in the set, while downweighting contributions from genes that do not track with other set members. And finally, mtDNA score was obtained by inputting core genes according to the corresponding code (doi:10.7150/thno.52717;doi: 10.1186/s12943-020-01170-0).

Statistical Analysis: The statistical methods used appear appropriate for the data type and research question. Nevertheless, the following points should be addressed:

**Q1.** Statistical Power: It would be beneficial to include a discussion on the statistical power of the study given the sample size and the number of variables being analyzed.

**Response:** Thank you for your suggestion. In the method, the statistical parameters are annotated.

**Q2.** Multiple Testing Correction: With multiple comparisons being performed, a correction method such as Bonferroni or False Discovery Rate (FDR) should be applied and reported.

**Response:** Thank you for your suggestion. In this study, FDR has been applied and reported (lines167 to 169).

**Q3.** Association Strength and Significance: The strength and significance of correlations should be reported comprehensively, including effect sizes and confidence intervals.

**Response:** Thank you for your suggestions. The strength and significance of correlations were reported comprehensively, and the effect sizes and confidence intervals have been added in the revised manuscript.

Results Interpretation: The findings of significant differences in mtDNA MRG expression between HCC samples and normal controls, as well as the identification of three molecular subtypes with distinct clinical and immune profiles, are intriguing. However, the interpretation of results should be cautious and conservative:

**Q1.**Biological Relevance: The biological implications of the molecular subtypes and their correlation with TME features need to be discussed in the context of existing literature.

**Response:** Thank you for your suggestions. The three molecular subtypes correspond to different classifications of the tumor microenvironment and have different biological processes and prognosis (lines 326 to 346).

**Q2.** Causal Inference: The observational nature of the study limits the ability to infer causality. Any discussion of causative mechanisms should be framed within this limitation.

**Response:** Thank you for your suggestions. All discussions of causative mechanisms were based on objective analysis of the expression of 22 mtDNA MRGs in HCC samples in public databases supported by existing articles (doi:10.3389/fcell.2022.785058;doi:10.1016/j.compbiomed.2023.106831;doi:10.1016/j.celrep.2016.12.019).

**Q3.** External Validation: Although not feasible in this study, future directions should include external validation of the mtDNA score model in independent cohorts.

**Response:** The lack of external validation is a shortcoming of this study. In the future, the existing results will be analyzed and some genes will be extracted for in vitro and in vivo experiments.

Response to Reviewer 2

**Reviewer 2**

Data section needs to be improved:1.Add more details regarding the batch correction method used.2. Include a figure showing data before and after batch correction (as supplementa-ry material).3.Specify the type of RNA data used (RNA-seq or microarray).4. Clarify the type of normalization applied to the data.

**Response:** Thank you for your suggestions.

1. Details regarding the batch correction method used in this study has been added, and changes in the revised manuscript were marked as red font.

2. A supplementary material has been provided, which including a figure showing data before and after batch correction (As shown in the following).

3. TCGA RNA sequencing data (FPKM format) were downloaded from the Genomic Data Commons. For the GSE76427 data from the Illumina platform, the normalized matrix file was directly downloaded.

4. The type of normalization that applies to the data has been clarified in the revised manuscript.

**Supplementary Fig. 1 Data set of HCC samples before and after batch correction.** A. Batch of HCC samples before correction. B. Batch of HCC samples after correction

The Method section needs to be improved. Authors mentioned the packages used without providing details about the methods. It should not be assumed that all readers are familiar with or can recall the methods implemented in each package (e.g., limma, ConsensusClusterPlus).

**Response:** Thank you for your suggestions. Details of the method have been added in the revised manuscript. If necessary, we will provide the corresponding code as a reference.

The clustering step using samples and genes is not clear and easy to follow. Please add details of the method, including the type of clustering (e.g., hierarchical clustering) and the distance metric used.

**Response:** Thank you for your suggestions. Details of the method have been added(lines 110 to 116, lines 139 to 143).

Additionally, add dendrograms to the heatmaps (e.g., Figures 2E and 5D) to better illustrate the clusters found and align with the findings.

**Response:** Thank you for your suggestion. Dendrograms reflect the similarities or correlations between members within a cluster. Figures 2E and 5D mainly showed the relationship between mtDNA MRGs molecular subtypes, genotypes and clinicopathological stages. Thus, there is no need to add dendrograms.

What does the y-axis label show in Figures 9B-E? Is it the IPS signature score? Additionally, there are more ICB signatures or biomarkers to consider, including PDCD1, CD274, PDCD1LG2, CTLA4, TIM3, LAG3, TIGIT, and resources like bhklab/SignatureSets: Compendium of published molecular signatures (github.com) and/or.

**Response: In** figures 9B-E, y-axis labels show the IPS signature scores of anti-PD-1 (ctla4-neg-pd1-pos), anti-CTLA-4(ctla4-pos-pd1-neg), or combined anti-CTLA-4/PD-1 (ctla4-pos-pd1-pos) immunotherapy. To promote understanding of tumor-immune cell interactions, Pornpimol Charoentong, et al characterized the intratumoral immune landscapes and the cancer antigenomes from 20 solid cancers and created The Cancer Immunome Atlas (https://tcia.at/). Using machine learning, they identified determinants of tumor immunogenicity and developed a scoring scheme for the quantification termed immunophenoscore (IPS). The IPS was a superior predictor of response to CTLA-4 and anti-programmed cell death protein 1 (anti-PD-1) antibodies in two independent validation cohorts. Therefore, we did not consider more ICB signatures or biomarkers (doi:10.1016/j.celrep.2016.12.019).
