# Supplementary material for: Correlation analysis of mitochondrial DNA maintenance-related genes with HCC prognosis, tumor mutation burden and tumor microenvironment features
Source: PLoS One. 2025 Jun 2;20(6):e0325033. doi: 10.1371/journal.pone.0325033 (PMC12129190; doi:10.1371/journal.pone.0325033)
Supplement: Attachment — Submitted filename: comments to the authers PONE-D-24-22922.docx [file pone.0325033.s003.docx]

**PLOS ONE**

**Correlation analysis of mitochondrial DNA maintenance-related genes with HCC prognosis, tumor mutation burden and tumor microenvironment features**

The authors addressed an interesting topic used appropriate methodology, highlighted the strengths of their study, and presented some interesting results and conclusions.

Overall, this is a clear, concise, and well-written manuscript. This article covers lots of information. On the one hand, many general statements are made without proper references. Please try to remove some of the general statements or speculations but add some details for some key refs for better illustration.

I have some concerns and remarks that I hope the authors can address to improve the paper

**The title and abstract** cover the central aspect of the work

But is better to have a graphical abstract to simplify the idea of the studied methodology

**-	The introduction** is relevant, and theory based. But:

Sufficient information about the previous study findings should be presented for readers to follow the present study rationale and procedures.

.

**- The methods** are generally appropriate, **-**

**- - Please add the** Data analysis and machine learning models

- I cant reach how These analyses were done and what was the used software version ?

How many machine learning models were used?

-how the performance of the oPLS-DA model was assessed ?

-

**the results** are clear, , the current study has innovations and advantages. However,

**-** the quality of the figures is poor and should be rectified ( very very poor)

**- please mention all abbreviations under the figures**

**-Points located far from the main cluster for their group may be potential outliers, indicating samples that do not follow the group trend. These samples should be investigated further, as they may represent unique biological conditions or data quality issues.**

**- Parameters for evaluation of each machine learning model discriminating pairs of groups should be clarified**

The performed study and results could have implication for research and clinical practice in the future. Therefore, the present article could be accepted for publication after revision and correction of the figures
