# Supplementary material for: Correlation analysis of mitochondrial DNA maintenance-related genes with HCC prognosis, tumor mutation burden and tumor microenvironment features
Source: PLoS One. 2025 Jun 2;20(6):e0325033. doi: 10.1371/journal.pone.0325033 (PMC12129190; doi:10.1371/journal.pone.0325033)
Supplement: Attachment — Submitted filename: Response_to_the_reviewers_auresp_3.docx [file pone.0325033.s004.docx]

**Dear Editor:**

Thank you very much for your efforts to review our manuscript entitled “**Correlation analysis of mitochondrial DNA maintenance-related genes with HCC prognosis, tumor mutation burden and tumor microenvironment features**”. We have diligently addressed all the comments, and incorporated appropriate revisions into the revised manuscript. The revisions have significantly strengthened the manuscript's scientific rigor and clarity. Enclosed below please find a point-by-point response document detailing how each suggestion has been implemented. The revised manuscript with all modifications clearly highlighted in red has been resubmitted to the system. We sincerely appreciate the reviewers' insightful feedback and trust that the revised manuscript now meets the journal's publication standards.

Yours sincerely,

Zhongji Meng

Department of Infectious Disease, Taihe Hospital, Hubei University of Medicine

No. 32. South Renmin Road, Shiyan 442009, China.

E-mail: [zhongji.meng@163.com](mailto:zhongji.meng@163.com)

**Additional Editor Comments:**

The authors addressed an interesting topic used appropriate methodology, highlighted the strengths of their study, and presented some interesting results and conclusions. Overall, this is a clear, concise, and well-written manuscript. This article covers lots of information. On the one hand, many general statements are made without proper references. Please try to remove some of the general statements or speculations but add some details for some key refs for better illustration.

I have some concerns and remarks that I hope the authors can address to improve the paper

The title and abstract cover the central aspect of the work

But is better to have a graphical abstract to simplify the idea of the studied methodology

Response: Thank you for your suggestions. The simplified graphical abstract is as follows:

The introduction is relevant, and theory based. But:

Sufficient information about the previous study findings should be presented for readers to follow the present study rationale and procedures.

**Response:** Thank you for your suggestions. Based on some of the investigators' previous studies (lines 84 to 93), we found that mitochondrial DNA maintenance-related genes (mtDNA MRGs) are closely related to the occurrence and progression of HCC. While the complex interactions among mtDNA MRGs in the development and progression of HCC are unclear, so that it’s necessary to investigate the mechanism of mtDNA MRGs in TME of HCC.

- The methods are generally appropriate,

- - Please add the Data analysis and machine learning models

- I cant reach how These analyses were done and what was the used software version ?

How many machine learning models were used?

**Response:** All the statistical analyses were performed using R version 4.2.2. In our study, Consensus Clustering and Principal Component Analysis (PCA) of unsupervised machine learning models are mainly used. In the analysis of Consensus Clustering, specific important parameters are set as follows: maxK=9, reps=50, pItem=0.8, pFeature=1.

-how the performance of the oPLS-DA model was assessed ?

**Response:**

**1. Data correction (before/after removal of batch effect)**

Problem: Different data sets (such as TCGA and GSE76427) have a "batch effect" due to differences in experimental conditions, resulting in false positive results.

The solution: Before correction: 89.7% explained by Dim1 (dominated by technical differences). After correction: Dim1 decreased to 18.9% (biological differences highlighted).

Significance: To eliminate technical interference and ensure that the differences found later truly reflect biological characteristics.

**2. Data integration (oPLS-DA score chart)**

Key points: Integrating multiple omics data (gene expression + clinical typing).

Graphic interpretation: Horizontal/vertical axis: principal components t1 (3%) and t2 (16%).

Sample distribution: TCGA (red) and GSE76427 (blue) partially overlap but are generally separable.

Significance: It proves that data from different sources can cooperatively reveal biological laws.

**3. Model efficacy (model efficacy -Rplot)**

Core indicators:

R2Y=0.767: The model can explain 76.7% of the sample classification differences.

Q2Y=0.672: 67.2% cross-validation prediction ability (>0.5 is valid).

p<0.05: The possibility of random guessing is excluded by substitution test.

Significance: The model is both reliable (high R2Y) and practical (high Q2Y).

**4. False positive control (replacement test chart)**

Method: Randomly scramble the labels 100 times and compare the real model (red line) with the random result (gray bar).

Result: The real R2Y/Q2Y was significantly higher than the random distribution (p=0.05).

No "virtual high" performance occurred.

Significance: Tat the differential genes and pathways discovered are not accidental.

In summary, our analytical process is rigorous (from data cleansing to model validation), and the biomarkers and typing results found have biological significance and potential clinical application.

**The specific flow chart is as follows**

A. Batch of HCC samples before correction. B. Batch of HCC samples after correction. C. Data integration (oPLS-DA score Chart). D. Model efficacy (model efficacy -Rplot).

the results are clear, , the current study has innovations and advantages. However,

- the quality of the figures is poor and should be rectified ( very very poor)

- please mention all abbreviations under the figures

**Response:** Thank you for your suggestions. All the figures have been refined and uploaded in the system. At the same time, all the abbreviations appearing in the figures have been fully expanded in their respective legends to ensure terminological clarity.

**-**Points located far from the main cluster for their group may be potential outliers, indicating samples that do not follow the group trend. These samples should be investigated further, as they may represent unique biological conditions or data quality issues.

**Response:** Thank you for your suggestions. These samples are all human specimens, individual differences are inevitable, while overall differences are within the acceptable range. On the other hand, TCGA and GEO data have been verified and used in many studies, so the data is relatively reliable. In our study, data correction has been carried out to remove technical interference and ensure that the differences found later reflect the biological characteristics.

- Parameters for evaluation of each machine learning model discriminating pairs of groups should be clarified.

**Response:** Thank you for your suggestions. In the manuscript, the evaluation parameters for each machine learning model have been annotated (Such as line 125, line 157, etc.)

**2nd set of questions:**

1. “Pornpimol Charoentong, et al analyzed tumor-immune cell interactions in 20 solid cancers, revealing the relationship between genotype and immunophenotype and created The Cancer Immunome Atlas (<https://tcia.at/>). Using machine learning, the determinants of tumor immunogenicity were identified and a quantitative scoring scheme called immunophenotypic scoring was developed.” Please add this explanation to the section titled “Analysis of cell differences in TME”.

**Response:** Thank you for your suggestions. We have added the above sentences “Pornpimol Charoentong, et al analyzed tumor-immune cell interactions in 20 solid cancers, revealing the relationship between genotype and immunophenotype and created The Cancer Immunome Atlas (https://tcia.at/). Using machine learning, the determinants of tumor immunogenicity were identified and a quantitative scoring scheme called immunophenotypic scoring was developed” to the section titled “Analysis of immune cell differences in the TME” (lines 140 to 145).

2. Lines 176-177 of revised manuscript: did you use the Draw Equation tool? Please put‘i’into subscript. Also, does the sigma have any initial and terminal values, i.e. i=0 .. n? Please start line 177 with “Where i represents the expression of mtDNA…”

**Response:** Thank you for your suggestions. We are very sorry for our carelessness. We have put‘i’ in the bottom right corner. i represents the expression of mtDNA phenotype-associated genes. The minimum value of ‘i’ is 0 and the maximum value of ‘i’ is 740.

3. In response to question #9, I do apologize, I stated my question incorrectly. I wanted to ask, how many genes do you increase the fold-change cutoff ? If the fold change is higher, you should get fewer genes.

**Response:** In our research, the P-value less than 0.001 was taken as the screening criteria, and the fold change of 1.5 was set as cutoff. If the fold change is higher, fewer genes will be screened out and some important genes with less changes will be filtered out.
